# Injectable Synthetic Beta-Tricalcium Phosphate/Calcium Sulfate (GeneX) for the Management of Contained Defects Following Curettage of Benign Bone Tumours

**DOI:** 10.3390/curroncol30040281

**Published:** 2023-03-27

**Authors:** Nima Razii, Laura M. Docherty, Mansur Halai, Ashish Mahendra, Sanjay Gupta

**Affiliations:** 1 Department of Trauma and Orthopaedics, Glasgow Royal Infirmary, Glasgow G4 0SF, UKsanjaygupta@doctors.org.uk (S.G.); 2 Division of Orthopaedic Surgery, St Michael’s Hospital, University of Toronto, Toronto, ON M5C 1R6, Canada

**Keywords:** benign bone tumour, curettage, bone defect, beta-tricalcium phosphate, calcium sulfate, synthetic bone graft

## Abstract

Benign and low-grade malignant bone tumours are often treated with curettage and filling of the resultant defect using any of a number of materials, including autologous bone grafts, allografts, or synthetic materials. The objective of this study was to report our experience using a synthetic bone graft substitute in these patients. Ten consecutive cases (four males, six females; mean age, 36 years) of benign bone tumours were treated surgically at a tertiary musculoskeletal oncology centre, between 2019 and 2021. Following curettage, the contained defects were managed with injectable beta-tricalcium phosphate/calcium sulfate (GeneX; Biocomposites Ltd., Keele, UK). The desired outcomes were early restoration of function and radiographic evidence of healing. No other graft materials were used in any of the patients. The mean follow-up was 24 months (range, 20–30 months). All patients in this series (100%) demonstrated radiographic evidence of healing and resumed their daily living activities. There were no tumour recurrences and no complications were encountered with the use of GeneX. In patients with contained defects following curettage of benign bone tumours, we found GeneX to be a safe and effective filling agent. These findings contrast with some existing studies that have reported local complications with the use of injectable beta-tricalcium phosphate/calcium sulfate.

## 1. Introduction

Benign and low-grade malignant bone tumours are typically treated with intralesional resection or curettage [1]. The resultant defect may be managed with a variety of materials, including autologous bone grafts, allografts, or synthetic materials, such as polymethylmethacrylate or composite substitutes. Autologous cancellous bone is widely regarded as the gold standard of graft material, being osteogenic, osteoinductive, and osteoconductive. However, problems such as limited graft size, wound complications, and persistent donor site pain have limited its use in clinical practice [2]. Concerns regarding the potential for immunogenicity or infection have likewise limited the use of demineralized freeze-dried allografts and xenografts [3]. Synthetic materials have therefore generated considerable interest in orthopaedics, and various bone graft substitutes are commercially available.

The objective of this study was to report our experience with the use of an injectable synthetic beta-tricalcium phosphate/calcium sulfate (GeneX; Biocomposites Ltd., Keele, UK) for the management of contained defects following curettage of benign bone tumours. The composite is manufactured through a process called zeta potential control [4], which produces a negative surface charge, and is suggested to provide both osteoconductive and osteoinductive characteristics. Contrasting reports on the efficacy and tolerability of GeneX to date have highlighted the need for further research.

## 2. Materials and Methods

In this study, 10 consecutive cases of benign bone tumours, which underwent curettage and grafting using an injectable synthetic beta-tricalcium phosphate/calcium sulfate (GeneX) at our institution between April 2019 and April 2021, were reviewed retrospectively. All patients were treated surgically under the supervision of two fellowship-trained orthopaedic surgeons specialising in musculoskeletal oncology (AM, SG). The study group comprised four males and six females, with a mean age of 36 years (range, 17–75 years) at the time of surgery. The median preoperative American Society of Anesthesiologists (ASA) physical status classification score was 1.5 (range, 1–2). For each patient, clinical records and radiographs were reviewed to ascertain the lesion site, tissue diagnosis, and outcome.

All patients underwent preoperative MRI as part of our multidisciplinary team (MDT) protocol and cases were subsequently discussed at regional meetings, which include input from an orthopaedic surgeon specializing in musculoskeletal oncology, a musculoskeletal consultant radiologist, and a clinical nurse specialist. Recommendations from the MDT were communicated to patients in the clinic, and written, informed consent was obtained prior to surgery in each case.

Intraoperatively, all patients receive a single dose of antibiotics on induction. The bone defect is prepared as per local protocol. After creating an osseous window, the tumour is curetted, and the base burred and irrigated with sterile water. These steps are repeated until only healthy bone is macroscopically visible. The defect is then dried and carefully filled with GeneX, which is prepared as per the manufacturer guidelines.

The grafted defect is then covered with a haemostatic foam sponge (Equispon; Equimedical BV, Zwanenburg, The Netherlands). Adjacent tissue is then carefully closed over the haemostat, to effectively create a seal over the graft. The remaining wound is closed in layers and a sterile dressing applied. Supplementary stabilization of the bone with metalware is not routinely performed and was not undertaken in this series. Surgical drains are not used, and patients do not typically require chemical thromboprophylaxis, as early mobilization is encouraged. Postoperative weight bearing status is determined on a case-by-case basis, considering tumour location and size.

Intraoperative tissue samples were sent for pathology, to confirm the histological tissue diagnosis for each patient. Data were collected on any postoperative complications, including adverse wound events. Patients were routinely followed up at 2 weeks postoperatively at our musculoskeletal oncology clinic and then at regular intervals thereafter, until the wound and X-ray appearances were satisfactory. We defined a postoperative surgical site infection (SSI) according to Health Protection Agency criteria up to 1 year following the index procedure, as synthetic graft materials are classified as medical devices.

## 3. Results

The clinical details of this case series are summarized in Table 1. The site of the lesion was the hand in four patients, the foot in three patients, tibia in two patients, and femur in one patient. The tissue diagnoses comprised enchondromas in four patients, aneurysmal bone cysts in three patients, fibrous dysplasia in two patients, and an epidermoid bone cyst in one patient. The mean lesion size, as determined by the largest diameter of the tumour on preoperative MRI, was 28.5 mm (range, 10–58 mm). At a mean follow-up of 24 months (range, 20–30 months), all patients in this series (100%) demonstrated radiographic evidence of resorption, and had resumed their daily living activities. The mean time to complete resorption was 12 months (range 7–19 months), but it is important to note that this was an approximation, contingent upon the availability of postoperative x-rays and the frequency of follow-up.

Patients with metatarsal lesions commenced heel weight bearing immediately after surgery in a forefoot off-loading shoe, whilst those with lesions in the hindfoot, femur, or tibia were allowed to toe-touch weight bear postoperatively, and gradually progressed to full weight bearing between 6 and 12 weeks. Patients with lesions in the hand were protected with a splint or plaster slab until the wound had healed, and then referred for hand physiotherapy between 4 and 6 weeks postoperatively to prevent stiffness. In this series, most patients had returned to work or achieved their baseline function between 12 and 16 weeks postoperatively.

**Figure 1:**
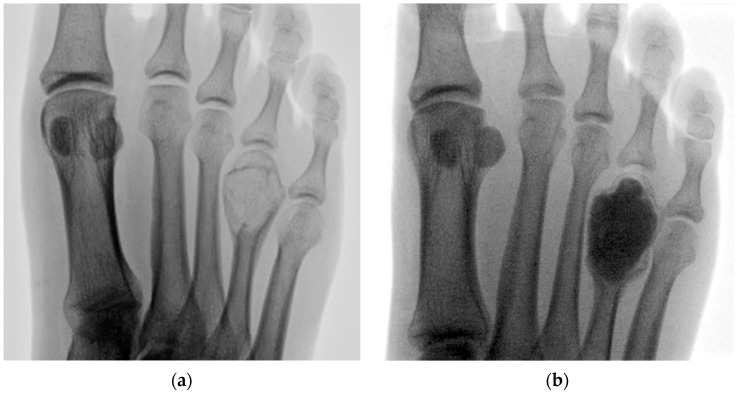

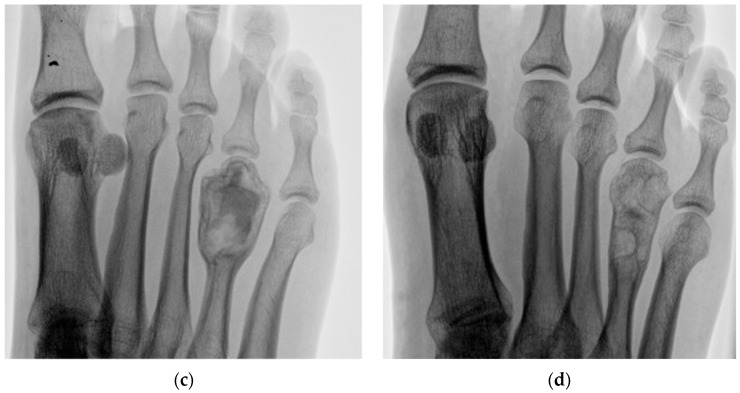
Dorsoplantar (DP) radiographs of right foot (*Case 1*): (**a**) preoperatively, showing an aneurysmal bone cyst in the distal fourth metatarsal; (**b**) immediately postoperatively, following excision and curettage, with the contained defect filled with GeneX; (**c**) at 6 months follow-up, the GeneX shows partial resorption and there is progression of bone healing with cortical definition maintained; and (**d**) at 12 months follow-up, the GeneX has been completely resorbed and there is evidence of significant bone remodelling.

**Figure 2:**
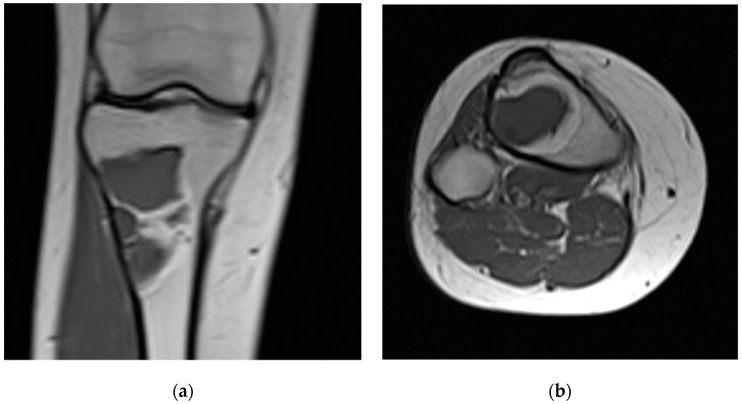

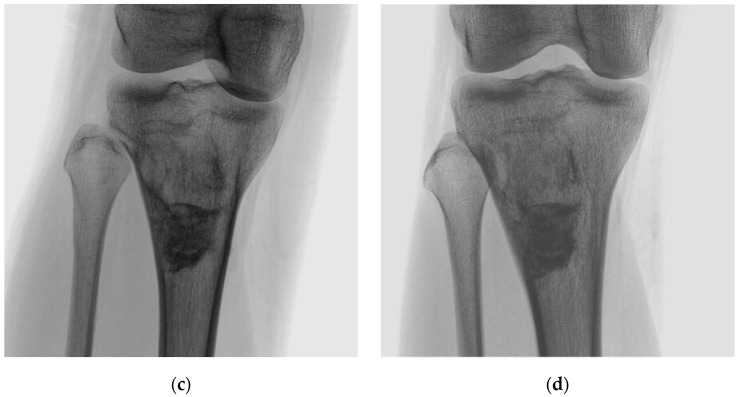
Preoperative coronal (**a**) and axial (**b**) T1-weighted MRI sequences, showing fibrous dysplasia in the right proximal tibial metaphysis (*Case 3*). Anteroposterior (AP) radiographs of right knee: (**c**) at 6 months follow-up, the GeneX shows partial resorption and the fenestration site is still visible on the lateral cortex; and (**d**) at 12 months follow-up, the GeneX continues to resorb, with progressive ossification of the defect, and the lateral cortex has healed.

**Figure 3:**
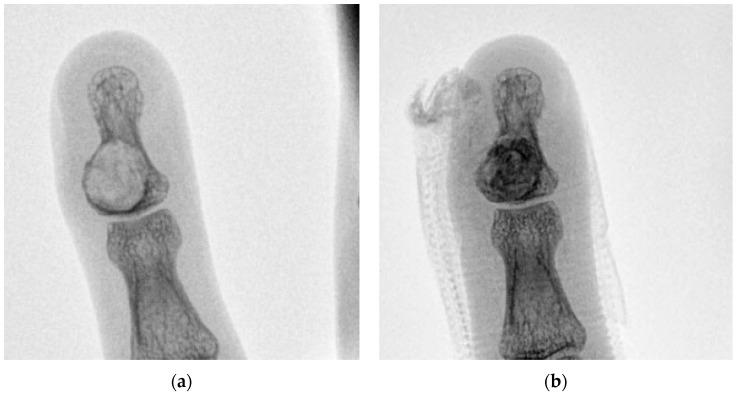

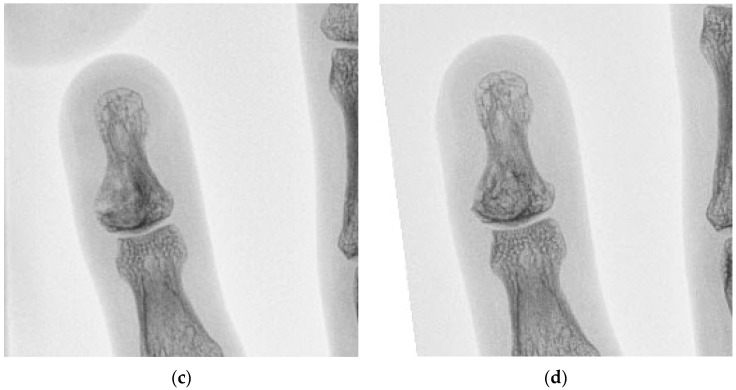
Posteroanterior (PA) radiographs of left little finger distal phalanx (*Case 5*): (**a**) preoperatively, showing an enchondroma at the base of P3; (**b**) immediately postoperatively, following excision and curettage, with the contained defect filled with GeneX; (**c**) at 6 months follow-up, the GeneX shows partial resorption and there is some evidence of bone healing; and (**d**) at 12 months follow-up, the GeneX has been completely resorbed and the affected cortex has largely remodelled.

There were no tumour recurrences in this cohort. There was one patient (Case 6) who remained under surveillance with MRI scans at 6-monthly intervals for 2 years postoperatively to exclude the possibility of local recurrence, and x-rays at most recent follow-up of 28 months were clear. No local or systemic complications were encountered with the use of GeneX, and none of the patients in this series developed an SSI. Figure 1 illustrates the example of Case 1, which involved an aneurysmal bone cyst in the foot. Figure 2 illustrates the example of Case 3, which involved fibrous dysplasia in the proximal tibia, and Figure 3 illustrates the example of Case 5, which involved an enchondroma in the hand.

## 4. Discussion

It is widely accepted that filling the resultant defect following curettage of benign bone tumours reduces the risk of pathological fractures, even in small volume defects, but there is no current consensus on which material is most suitable in clinical practice. Whilst an autologous graft has the best properties for integration, a recent systematic review indicated that it was associated with the highest rate of complications, after leaving the defect unfilled, predominantly due to donor site pain [5]. In addition to the overall complication rate, the infection rate was also higher in patients who received autologous graft (1.05%), than either PMMA (0.70%) or bone substitute (0.98%), although not as high as the allograft group (2.32%).

Synthetic graft materials may be classified within two broad groups, calcium phosphate and calcium sulfate. Calcium phosphate minerals comprise hydroxyapatite, which demonstrates minimum resorption in vivo, in addition to tricalcium phosphate and tetracalcium phosphate, which are generally resorbed over a period of several months [6]. Calcium sulfate products tend to resorb much faster, over a period of several weeks, and are typically derived from the processing of gypsum, although fully synthetic calcium sulfate is also commercially available [7].

A comparative retrospective cohort study between 13 patients who received autologous bone grafting and 11 patients who received beta-tricalcium phosphate following curettage of hand enchondromas reported equivalent radiological and functional outcomes between both groups [8]. However, there was a significantly reduced surgical time in the bone substitute group (25 min mean difference), with a 30.8% rate of persistent donor site pain in the autologous bone graft group. The authors emphasized the importance of avoiding graft spillage into the surrounding soft tissues, and “sealing the window” with cortical bone following curettage—or fibrin glue where this was not possible—in order to contain the defect. In our cohort, a collagen-based membrane was used as an alternative.

Beta-tricalcium phosphate in particular has gained popularity over hydroxyapatite in recent years as a bone graft substitute, due to its excellent biocompatibility and osteoconductivity [6,9]. A case series of 53 patients, describing the use of purified beta-tricalcium phosphate following curettage of benign bone tumours, reported excellent outcomes in terms of safety and tolerability, with no adverse reactions, and complete resorption in 23 cases (43%) by 26 months [10]. These findings were largely in keeping with a study from France, which reported on the outcomes of synthetic beta-tricalcium phosphate for a wide variety of indications in 110 patients [9]. There were no poor results or infections in any of the 14 individuals who had been treated for benign bone tumours.

A more recent study by Chung et al. [11], in which 20 patients with benign bone tumours underwent curettage and cavity filling with beta-tricalcium phosphate granules, reported complete resorption and bone remodelling in 55% of the cohort by 12 months, whilst the remaining 45% had shown partial resorption by 24 months. Interestingly, the authors did not find any association between the filling volume and time to resorption, which was in contrast to an earlier study by Nicholas and Lange, using beta-tricalcium phosphate granules for the same indication in 18 participants, which concluded that healing was dependent upon the defect size [12].

A number of studies have investigated the use of injectable calcium sulfate as a bone graft substitute. In a randomized controlled trial of 56 patients who underwent curettage and graft filling of a contained defect, there were no significant differences in outcomes between patients that received injectable calcium sulfate and those who received demineralized bone matrix [13]. Another case series of 46 patients, involving the use of injectable calcium sulfate for the same indication, reported excellent functional results and complete resorption in 83% of the cohort by 24 months [14].

Biphasic and triphasic composites have been developed in order to combine the benefits of rapid osteoconductivity of calcium sulfate with the compressive strength and slower, cell-mediated resorption of calcium phosphate [15,16]. Fillingham et al. retrospectively reviewed 46 patients who received a composite graft, and reported surgical site infections (SSI) in two patients (4%), but graft removal was not required in either case, and most patients returned to normal function [17]. Tan et al. performed a radiological review of 25 patients who received the same material and observed complete resorption in 64% of the cohort by 12 months [18], which compares favourably with some of the earlier studies describing the use of beta-tricalcium phosphate alone [10,11], although it is difficult to adjust for varying defect sizes filled in each study.

GeneX has generated controversy in the published literature. In a series of 31 patients who received between 5–60 cc of GeneX after curettage of benign and low-grade malignant bone tumours, Friesenbichler et al. reported adverse reactions in 16% of their cohort [19]. Unlike the present study, an allograft was used in two patients, both of whom experienced complications, and the authors subsequently clarified that “sealing” or closure of the bone window was not possible in most cases [20]. More recently, Lowery et al. described the use of GeneX for managing periarticular bone loss in 40 tibial plateau fractures, in which one instance of postoperative wound leakage was identified (2.5%), and no other complications were related to the graft material [21]. Shin et al. reported no complications related to the use of GeneX for augmenting the cephalomedullary fixation of 115 patients with pertrochanteric hip fractures [22]; similarly, Sung et al. reported no increase in adverse events when GeneX was used for the same indication in 41 patients [23]. In both of these studies, inherent containment was achieved, as GeneX was injected into the femoral head and neck.

Although very good results were reported in both an animal model and 38 patients who underwent percutaneous vertebroplasty for trauma [24,25], adverse events were described in a separate report of three patients who had received between 5–10 cc of GeneX directly adjacent to soft tissues of the cervical and thoracic spine following decompression and fusion [26]. The authors concluded that it should not be placed in direct contact with soft tissues, which reinforces earlier suggestions that following the use of injectable calcium phosphate-based bone substitutes, the filled defect should be contained [27]. Indeed, this was considered an important aspect of our surgical technique, where grafting and containment of the defect was carefully undertaken in each case to prevent extrusion of the graft material into the surrounding soft tissues. None of the patients in our case series experienced an adverse wound event.

Aneurysmal bone cysts (as seen in Cases 1, 6, and 8) comprise around 1% of all bone tumours, and typically present in the metaphyses of long bones in patients under 30 years old [28]. Although benign, they are locally aggressive lesions, and curettage—with the option of grafting the resultant defect—remains the standard of care. Local recurrence is considered the predominant difficulty in the management of aneurysmal bone cysts, and adjuvant therapies (such as phenol or cryotherapy) are available [29], but were not used in our patients. Gibbs Jr. et al. advocated the use of a high-speed burr during curettage [30], which was routinely performed for all patients in this study. They achieved a success rate of 90% in resecting aneurysmal bone cysts with surgery alone, and found that all local recurrences occurred within 2 years of the index procedure in skeletally immature patients. In this study, we kept one patient (Case 6), who was considered at a higher risk of recurrence, under surveillance with interval MRI scans, which have remained clear.

Enchondromas (as seen in Cases 2, 4, 5, and 10) consist of hyaline cartilage and involve up to 10% of all bone tumours. They typically present as solitary lesions in the metaphysis of bones in the hands and feet in young adults, although occurrence in the distal femur is also well recognized [31]. They are usually asymptomatic, but expansion may cause mechanical pain or pathological fractures. The standard of treatment for symptomatic lesions is curettage and, although defect management varies widely, the recurrence rate is much lower than with aneurysmal bone cysts [32,33]. Enchondromas have the potential for malignant transformation to secondary chondrosarcoma, but this is rare in patients with solitary lesions [34].

Fibrous dysplasia (as seen in Cases 3 and 9) comprises less than 1% of all bone tumours, and is characterized by a fibro-osseous mass, which lacks a normal trabecular pattern, and endosteal scalloping without periosteal reaction [35]. Monostotic fibrous dysplasia is the more common form of the disease, typically affecting patients between 10 to 30 years old, whilst polyostotic fibrous dysplasia tends to present in younger children and may be associated with genetic conditions, such as McCune-Albright, Jaffe-Lichtenstein, and Mazabraud syndromes [36,37]. Monostotic fibrous dysplasia usually occurs in the craniofacial bones, ribs, femur, or tibia (Figure 2). Asymptomatic cases are usually managed conservatively, whilst surgery is performed for indications such as mechanical pain, progressive deformity, pathological fractures, cystic degeneration, or malignant transformation (which is very rare, but more common in Mazabraud syndrome, and cases which have been treated with radiotherapy) [35,38,39,40]. The recurrence rate of fibrous dysplasia in long bones following curettage and grafting remains unclear, although skeletally mature patients and those with monostotic lesions appear to have a better prognosis [40,41]. The use of graft materials with slower resorption rates—such as beta-tricalcium phosphate or cortical allograft—has also been advocated for these cases [10,42].

Intraosseous epidermoid cysts (as seen in Case 7) are rare but well-described benign bone tumours that usually involve the skull or terminal phalanges [43]. Although their aetiology is uncertain, it has been suggested that they be congenital, iatrogenic, or traumatic, involving fragments of epidermal tissue entering through a cortical breach and subsequently proliferating [44]. Enchondromas are much more common tumours than epidermoid bone cysts, but tend to present in the proximal metaphyseal segments of the phalanges (Figure 3), at the site of growth plate ossification [45]. The recurrence of intraosseous epidermoid cysts is extremely unlikely following curettage and excision of the cyst capsule, irrespective of whether bone grafting is performed [46].

We recognize that this study has limitations. First, in keeping with the existing literature, it has a retrospective design and describes a limited group of patients with various tissue diagnoses. Second, our cohort did not involve any patients with significant co-morbidities, but benign bone tumours most commonly present in young patients. However, the use of GeneX in elderly trauma patients (with higher ASA scores) has previously been reported [22]. Third, although the objective of our study regarding the use of injectable synthetic beta-tricalcium phosphate/calcium sulfate was achieved, longer follow-up would be useful to identify any tumour recurrences. Nevertheless, there was no loss to follow-up at a mean of 2 years, and no cases of recurrence within the study period. Finally, although lacking a control group for comparison, we have presented consecutive cases treated with the same surgical technique and contained defect management using a single injectable composite, without the use of other graft materials.

## 5. Conclusions

Whilst the applications of GeneX as a bone graft substitute for trauma procedures have been encouraging, this study may help to guide its use in the context of benign bone tumours. In our experience, when patients are managed with an MDT approach, and the surgical technique involves meticulous curettage and containment of the bone defect, we found GeneX to be a safe and effective filling agent.

## Figures and Tables

**Table 1:** Clinical details of patients managed with GeneX.

Case	Age/Sex	ASA	Lesion Site	Diameter (mm)	Tissue Diagnosis	Follow-Up (Months)
1	23 M	2	Right foot 4th metatarsal	20	Aneurysmal bone cyst	30
2	34 M	1	Left middle finger P1	32	Enchondroma	24
3	17 F	1	Right proximal tibia	58	Fibrous dysplasia	22
4	27 F	2	Left foot 2nd metatarsal	24	Enchondroma	23
5	36 M	1	Left little finger P3	10	Enchondroma	25
6	25 F	2	Right os calcis	33	Aneurysmal bone cyst	28
7	71 M	2	Right middle finger P3	20	Epidermoid cyst	27
8	23 F	1	Left hand 2nd metacarpal	27	Aneurysmal bone cyst	23
9	27 F	1	Left tibial diaphysis	41	Fibrous dysplasia	23
10	75 F	2	Right medial femoral condyle	20	Enchondroma	20

## Data Availability

The data supporting the findings of this study are available within the article. Further inquiries can be directed to the corresponding author.
